# Reconstruction of Upper and Lower Limb Defects with Medial Sural Artery Perforator Flaps: Is Aesthetics Worth the Effort? A Retrospective Analysis

**DOI:** 10.1055/s-0043-1770956

**Published:** 2023-07-10

**Authors:** Ziyad Alharbi, Sarah Qari, Faris Almarzouqi, Khalid Khatib, Savas Tsolakidis, Anas Fathuldeen, Gerrit Grieb, Hans-Oliver Rennekampff

**Affiliations:** 1 Plastic Surgery and Burn Unit, Dr. Solaiman Fakeeh Hospital, Jeddah, Saudi Arabia; 2 Clinical Sciences Department, Fakeeh College for Medical Sciences, Jeddah, Saudi Arabia; 3 Department of Plastic Surgery, International Medical Center, Jeddah, Saudi Arabia; 4 Department of Plastic Surgery, Saudi German Hospital, Jeddah, Saudi Arabia; 5 Department of Plastic Surgery and Hand Surgery, Burn Center, Medical Faculty, Austrian Cluster of Tissue Regeneration, Ludwig Boltzmann Institute for Experimental and Clinical Trauma, RWTH Aachen University Hospital, Aachen, Germany; 6 Department of Surgery, Plastic Surgery College of Medicine, Hail University, Hail, Saudi Arabia; 7 Department of Plastic Surgery and Hand Surgery, Burn Center, Medical Faculty, RWTH Aachen University Hospital, Aachen, Germany; 8 Department of Plastic Surgery and Hand Surgery, Gemeinschaftskrankenhaus Havelhoehe, Berlin, Germany; 9 Department of Plastic and Aesthetic Surgery, Burn Surgery, Rhein-Maas Klinikum, Würselen, Germany

**Keywords:** free flaps, medial sural artery perforator flaps, fasciocutaneous flaps, reconstruction, hand deformity, foot deformity, extremity

## Abstract

**Background** One of the most essential goals in managing complex limb defects is obtaining adequate soft tissue coverage with excellent functional and aesthetic outcomes. Free perforator skin flaps represent an optimal option for such defects. Therefore, our intention was to reconstruct these kinds of defects with thin fasciocutaneous flaps without the need for debulking. Herein, we define the legitimate use of the medial sural artery perforator (MSAP) flaps for small-moderate size defect coverage of the hand and foot.

**Patients and Methods** Seven patients received MSAP flaps for reconstruction of different hand and foot defects, of which the majority were males (4/7). Age, sex, flap size, location, number of perforators, recipient vessel, type of anastomosis, technique of donor site closure, and postoperative morbidity were recorded. Patients' age ranged from 48 to 84 years.

**Results** Single-stage debridement followed by reconstruction was performed. Flap sizes ranged from 6 to 18 cm in length and 4 to 10 cm in width. The pedicles of 6 flaps were anastomosed to the tibial artery system (three posterior tibial artery, three dorsalis pedis artery) and one to the ulnar artery.

**Conclusion** MSAP flap can be a versatile option for single-stage reconstruction of small-moderate size defects of the extremities, where thin, soft tissue envelope is required. This flap has lower donor site morbidity, more tedious elevation process, and has a good reconstructive and aesthetic result without the need for debulking in the future.

Reconstruction of upper and lower extremity defects remains challenging for orthopaedic and plastic surgeons. 1 2 3 4 One of the most important goals in managing complex limb defects is obtaining adequate soft tissue coverage with excellent functional results and an acceptable aesthetical outcome. Free perforator skin flaps represent an optimal option for such defects. 2 3 4 5 6

However, reconstruction of soft tissue defects in the lower and upper limbs still poses a considerable challenge for the reconstructive microsurgeon nowadays, especially in terms of aesthetical reconstruction. The choice of flap depends mainly on the type of defect, the surgeon's preference, expertise, and the availability of flap donor areas.

Taylor and Daniel described in 1975 a “popliteal island flap” supplied by the musculocutaneous branches of the medial and lateral sural vessels. 7 In 1996, Montegut and Allen presented, based on their anatomical studies of the calf, the sural artery perforator flap, primarily as a local alternative to the gastrocnemius flap. 8 The free medial sural artery perforator (MSAP) flap was first described in 2001 by Cavadas et al. 9 It has since been described for both upper and lower extremities as well as head and neck reconstruction. Since then, other studies have assessed the anatomical basis of the MSAP flaps and their clinical versatility.

This flap has gained popularity and has become one of the free flap choices for reconstructing small-to-medium sized defects in the hand and foot region. Notwithstanding, many surgeons stated the difficult dissection of its perforator and possible anatomical variations of the main pedicle that raised the main drawbacks of this flap. 10

Considering the actual detailed knowledge of the calf perforator anatomy and possessing adequate microsurgical expertise, this flap can safely be raised and transferred, especially in thin patients presenting with small-to-moderate size soft tissue defects, where thin pliable skin is needed to achieve optimal postoperative aesthetic and functional results. Recently, the MSAP flap has been in intense discussion for its legitimate use in reconstruction of the distal lower extremity, especially where other options may exist, such as free anterior lateral thigh (ALT) flaps and others. 10 Also, the decrease in donor site morbidity always plays a role for its choice.

Our intention was to reconstruct these kinds of defects with thin fasciocutaneous flaps without the need for debulking in the future. Herein, we define the legitimate use of the MSAP flaps for small-to-moderate size defect coverage of the hand and foot.

## Patients and Methods

This is a retrospective analysis study for seven consecutive patients received MSAP flaps for reconstruction of different hand and foot defects, of which the majority were males (four out of seven). Age, sex, flap size, location, number of the perforators, recipient vessel, type of anastomosis, technique of donor site closure, and postoperative morbidity were recorded as shown in Table 1 . Patients' age ranged from 48 to 84 years. Written informed consent for participation in the study was obtained from participants or the participant's guardian.

**Table 1:** Summary of case series patients

Case no.	Sex and age (y)	Size of flap (length in cm × width in cm)	Flap location	Perforators number	Recipient vessel	Anastomosis type	Donor site closure	Complications closure
1	F, 67	10 × 7	Hand	2	Ulnar artery	E2S	Direct closure	
2	F, 59	6 × 5	Foot	1	DPA	E2S	Direct closure	–
3	M, 84	12 × 5	Foot	1	PTA	E2S	Direct closure	–
4	M, 48	15 × 10	Foot	2	PTA	E2S	STSG	Vein thrombosis which has been revised
5	M, 52	7 × 6	Foot	1	PTA	E2S	STSG	–
6	F, 58	6 × 4	Foot	1	DPA	E2S	Direct closure	–
7	M, 53	18 × 9	Foot	1	DPA	E2E	Direct closure	–

Abbreviations: DPA, dorsalis pedis artery; E2E, end to end; E2S, end to side; f, female; M, male; PTA, posterior tibial artery; STSG, split-thickness skin graft.

**Fig. 1:**
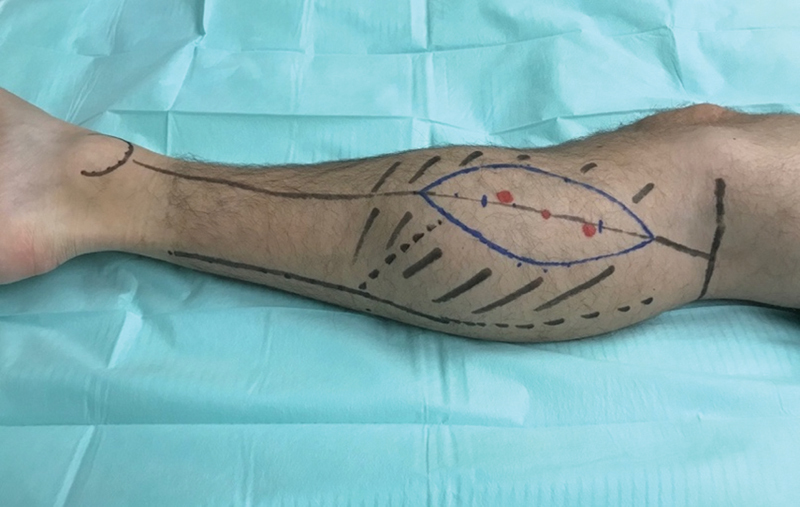
Markings for the free medial sural artery perforator (MSAP) flap.

A hand-held Doppler probe was used to map out the perforators from the medial gastrocnemius ( Fig. 1 ). The main preferred perforator was located 8 cm distal to the popliteal crease located on a line from the middle of the popliteal crease to the medial calcaneus. If more than one perforator was present, they were usually located 14 to 16 cm distal to the popliteal crease. The anteromedial border of the flap is elevated to confirm the location and size of the perforators. When one or two sizable perforators are identified, the opposite border is then incised, and the flap is elevated. The pedicle is then freed from the medial head of the gastrocnemius muscle through an intramuscular dissection. Hemostasis was meticulously performed using bipolar cautery. Basically, the course of the main trunk of the vascular pedicle is parallel to the gastrocnemius muscle fibers and can be exposed by splitting them. The pedicle may be dissected 8 to 15 cm in length depending on the site of the skin perforators. When dissecting the flap, it is very important to decide either to perform a suprafascial or subfascial dissection. A subfascial dissection will ensure more blood supply to the flap but, on the other hand, it might cause more morbidity and damage to the muscle. Hence, we preferred a suprafascial dissection, but this requires meticulous dissection under magnification loops. If two perforators are present, then a clear cut on the fascia should be made to identify the connecting vessel going to the pedicle. In this regard, the nerve should also be identified and dissected to protect it. Donor sites were closed primarily, which was possible in all cases except two, which were large with a width of 8 to 10 cm. In such cases, a split-thickness skin graft was used to close the donor site.

## Results

Debridement followed by reconstruction was performed in all patients. Flap sizes ranged from 6 to 18 cm in length and 4 to 10 cm in width. The pedicles of three flaps were anastomosed to the posterior tibial artery, three flaps to the dorsalis pedis artery, and one flap to the ulnar artery.

Primary donor site closure was performed in five out of seven patients, while split-thickness skin grafting was applied in two out of seven patients. In the intraoperative setting, free MSAP flaps with one (five out of seven patients) or two (two out of seven patients) perforators have been found ( Table 1 ).

The average operation time was 240 minutes, including an average flap harvest time of 120 minutes. All flaps survived, while one complication occurred: venous congestion due to acute thrombosis of the venous anastomosis in one of the flaps inserted for a foot defect. The initial anastomosis was done with the posterior tibial artery (E2S) and the venae comitantes. Once venous congestion and thrombosis were suspected, the patient was taken directly to the operating room for revision and venous thrombosis was clearly identified. Reanastomosis was then achieved by using another proximal vein branch close to the flap, while ensuring there was enough distance to reach the flap's vein without causing tension. After this anastomosis the circulation of the flap was completely intact. The average hospital stay was 14 days. Once complete wound healing was achieved, patients with lower limb defects were initially fitted with orthopaedic shoes to prevent eventual pressure sores around the nonsensate areas of the flaps and returned to normal ambulation within 2 months postoperatively.

No flap adjustments or defatting were employed. The total clinical follow-up time ranged from 10 to 20 months in which clinical analysis and examination was done to ensure there was no flap or donor site morbidity, with no patient lost to follow-up.

## Cases Examples

### Case 1

**Fig. 2:**
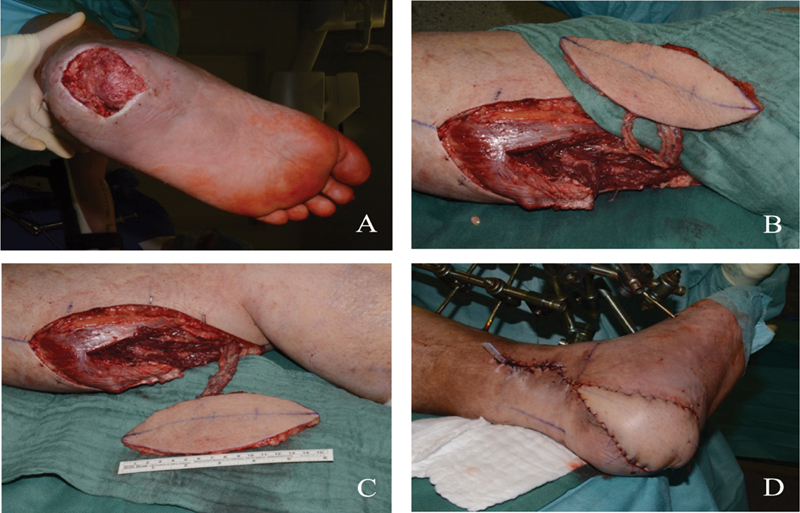
( **A** ) Degloving injury with bone exposed defect on the heel region. ( **B** ) Free medial sural artery perforator (MSAP) flap has been harvested and intraoperative view after dissection with two perforators to the medial sural pedicle. ( **C** ) Free MSAP flap has been harvested and intraoperative view after dissection with two perforators to the medial sural pedicle is shown. ( **D** ) The defect has been closed with the flap through “end to side” anastomosis with the posterior tibial artery.

This is a 48-year-old male who sustained a degloving injury with an exposed bone defect on the heel region through high-energy trauma, leaving a complex soft tissue defect ( Fig. 2A–D ). Free MSAP flap has been harvested, and the intraoperative view after dissection with two perforators to the medial sural pedicle is shown ( Fig. 2B, C ). The defect has been closed with the flap through “end to side” anastomosis with the posterior tibial artery and venae comitantes ( Fig. 2D ).

### Case 2

**Fig. 3:**
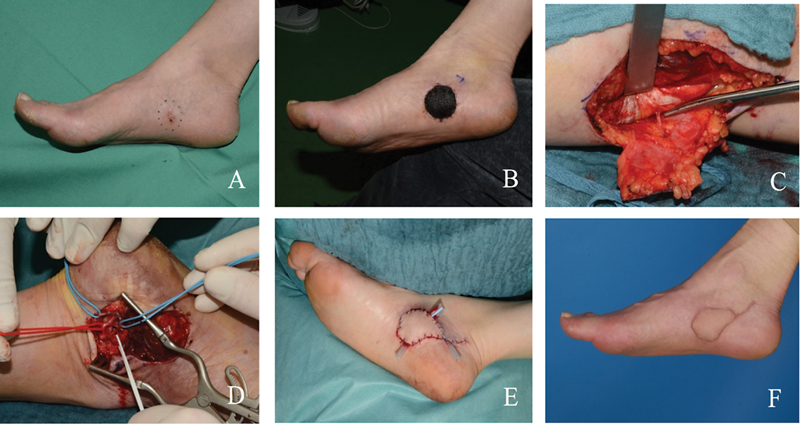
( **A** ) Superficial spreading malignant melanoma in the medial side of the foot sole. ( **B** ) Soft tissue defect has been resulted after resection of the tumor with 1 cm safety margin. ( **C** ) Intraoperative view after dissection of the medial sural artery perforator (MSAP) is shown here. ( **D** ) Preparation of recipient vessels in the foot (posterior tibial artery and veins). ( **E** ) The defect has been closed with MSAP flap. ( **F** ) Appearance of the flap site 6 months postoperatively.

This is a 58-year-old female with superficial spreading malignant melanoma in the medial side of the foot sole ( Fig. 3A–F ). Soft tissue defect resulted after resection of the tumor with a 1-cm safety margin ( Fig. 3B ). The intraoperative view after the dissection of the MSAP is shown in Fig. 3C . Furthermore, the preparation of the recipient vessels in the foot is also demonstrated ( Fig. 3D ). The defect has been closed with the flap through “end to side” anastomosis with the posterior tibial artery ( Fig. 3E ). The last picture shows the appearance of the flap site 6 months postoperatively ( Fig. 3F ).

### Case 3

**Fig. 4:**
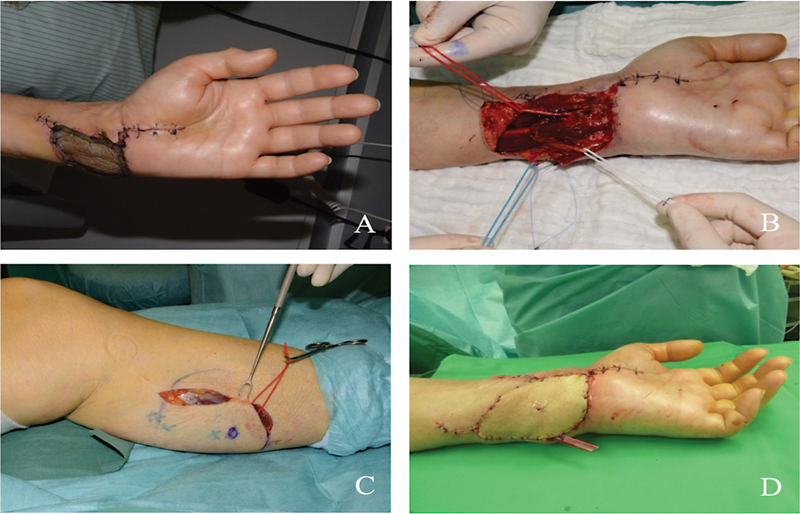
( **A** ) Soft tissue defect with exposed tendons. ( **B** ) Preparation of recipient vessels (ulnar artery and veins) has been performed during harvesting of the flap. ( **C** ) Harvesting of medial sural artery perforator (MSAP) flap with two perforators. ( **D** ) The defect has been closed with MSAP flap through “end to side” anastomosis with the ulnar artery.

This is a 67-year-old female with a defect on the hand region after trauma, leaving a soft tissue defect with exposed tendons ( Fig. 4A–D ). She was referred from the trauma and orthopedic department after stabilization for soft tissue defect reconstruction. Preparation of the recipient vessel (ulnar artery) has been performed during harvesting of the flap ( Fig. 4B ). The defect has been closed with the MSAP flap through “end to side” anastomosis with the ulnar artery ( Fig. 4C ). The last picture shows the appearance of the flap after microsurgical anastomosis ( Fig. 4D ).

### Case 4

**Fig. 5:**
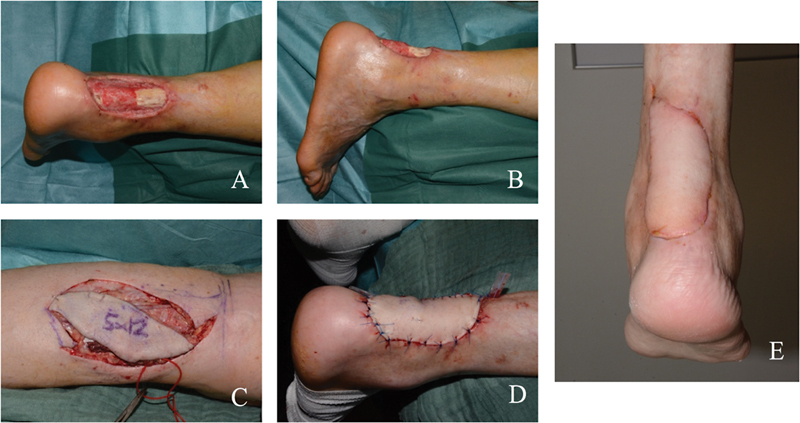
( **A** ) Soft tissue defect on the Achilles tendon after trauma, leaving a complex soft tissue defect. ( **B** ) Lateral view of the defect. ( **C** ) Free medial sural artery perforator (MSAP) flap has been harvested. ( **D** ) The defect has been closed with the flap through “end to side” anastomosis with the posterior tibial artery. ( **E** ) The last picture shows the appearance of the flap site 6 months postoperatively.

This is an 84-year-old male with a complex soft tissue defect on the Achilles tendon resulting from trauma which did not heal for a long time resulting in chronic wound formation, thus we received the patient for soft tissue reconstruction ( Fig. 5A, B ). Free MSAP flap has been harvested, and an intraoperative view is shown here after dissection of the perforators to the medial sural pedicle ( Fig. 5C ). The defect has been closed with the flap through “end to side” anastomosis with the posterior tibial artery ( Fig. 5D ). The last picture shows the appearance of the flap site 6 months postoperatively ( Fig. 5E ).

## Discussion

Success in soft tissue reconstruction mainly depends on the defect's size, depth, and location, the flap options, and the correct three-dimensional application of the flap. Reconstruction of hand and foot defects is a demanding task which aims to restore not only the function but also the aesthetics and ideal form.

Kao et al compared the results of the radial free forearm flap with the MSAP flap, and they found a 100% survival rate in both groups. 11 As expected, the subjective outcome regarding donor site morbidity was superior for the MSAP flap. Kao et al later described a case series of 26 MSAP flaps (an extension of their previous publication) for head and neck reconstruction, of which one flap failed 2 days postoperatively because of venous insufficiency. 12

One ongoing controversy discusses whether the MSAP flap justifies its harvesting from the ipsilateral region, where a defect-dependent disability is already preexistent, over the use of other flaps harvested from the groin or thigh (e.g., the superficial circumflex iliac artery perforator flap, medial circumflex femoral artery perforator flap, or ALT flap), which accounts for a lower donor site morbidity and can provide an equal or more flap thickness and size. 13

Chen et al described their series of MSAP flaps for distal limb defects of 11 patients, in which one flap failed. 14 The failed flap developed venous congestion and could not be salvaged. Kinking of the perforator or the small diameter of the perforator was suspected to be the underlying reason for failed venous drainage. Wang et al presented their case series of MSAP flaps for distal limb reconstruction, including 34 flaps of which 5 underwent partial necrosis. 15 Based on their description, the partial failure of the cases was due to a venous problem (partial purpling with bubbles and subcutaneous hematocele), which was handled conservatively with regular wound management. Kim et al also described their series of patients with plantar defects reconstructed with the MSAP flap. 16 Eleven patients were included, and all flaps survived. However, one flap developed venous congestion but was managed with leech therapy. Hallock has even performed 14 MSAP flaps for ipsilateral lower extremity defects, in which one failed because of venous congestion and was salvaged with another free flap. 13 Only two case series did not have any venous failures; instead, each had one arterial flap failure. He et al presented their case series of nine patients where they used the MSAP flap for oral reconstruction using preoperative computed tomography angiography (CTA) planning. 17 They found a good correlation between the locations of the perforators when comparing the CTA with intraoperative findings. One case failed because of arterial insufficiency 60 hours postoperatively, which could not be salvaged.

The MSAP flap has also garnered attention for both upper and lower extremity reconstructions. Lin et al described a series of 14 patients where the MSAP flap was used for hand reconstruction. 18 Only one flap failed, which was attributed to a significant difference in donor and recipient artery diameter (1:4) and vasospasm. No cases with venous problems were described.

In our practice, ALT flaps are still the primary option for skin flaps. However, if the latter cannot be harvested for a specific reason or if patients wish for a thinner flap, then the MSAP flap would be the alternative choice. Another important fact is that we gain more confidence with these types of flaps, especially for the hand and foot region. This can be justified as the MSAP flaps are thin in nature, providing good functional and aesthetical outcomes. Furthermore, defects on the hand and foot usually do not need long pedicle flaps for anastomosis as the recipient anastomosis site is almost close to the defect with different arterial options. The advantages of the MSAP flap include the possibility of a two-team approach as the flap can be harvested with the patient in the supine position, with the hip abducted and the knee flexed. The flap donor site can be closed directly if the width is not too large (our limit has been 9 cm in width for direct closure). We have experienced that the pedicle is of sufficient length, and the vessels mostly have a suitable diameter. No major vessels are sacrificed during this operation.

MSAP flap can be a versatile option for single-stage reconstruction of small-to-moderate sized defects of the upper and lower extremity, where thin, soft tissue envelope is required. This flap has lower donor site morbidity, more tedious elevation process, and has an excellent reconstructive and aesthetic result without the need for debulking in the future. Although MSAP flaps are relatively thin, it is important to note that they could vary in thickness depending on the body mass index (BMI) and sex of the patient, particularly in females with a higher BMI. 19 Scarring in this area could also pose a problem, especially in females when large flaps would require skin grafting or when the high tension area could result in widening of the scar when primary closure was possible. Lastly, the risk of flap failure due to late-onset venous thrombosis seems to be somewhat higher than the more established free flaps. Therefore, this flap should be monitored more closely for venous problems. For our patients the average length of stay in the hospital was 14 days. This was to provide the patient with anticoagulation therapy in the ward as well as continuous monitoring of the flap and donor site for early detection of vascular compromise. Usually, 7 to 10 days is sufficient for neovascularization, the longer stay for some of our patients was due to other comorbidities being treated by other departments. Given the low morbidity of the flap and the ideal characteristic of the tissue, this could very well become the flap of choice for hand and foot reconstructions.

Limitations in our article included the small number of patients and heterogeneous etiology of the defects.

## Conclusion

The MSAP flap can be a versatile option for single-stage reconstruction of small-to-moderate size defects of the upper and lower extremity, where thin, soft tissue envelope is required. This flap has lower donor site morbidity, more tedious elevation process, and has a good reconstructive and aesthetic result without the need for debulking in the future.
